# Treatment of advanced medullary thyroid cancer

**DOI:** 10.1186/1756-6614-6-S1-S7

**Published:** 2013-03-14

**Authors:** Johannes Smit

**Affiliations:** 1 Department of General Internal Medicine, Radboud University Nijmegen Medical Centre P.O. Box 9101, 6500 HB Nijmegen, The Netherlands

## Abstract

Therapy decisions in advanced medullary thyroid carcinoma should be guided by a critical appraisal of the natural disease course (slowly progressive vs. aggressive) and benefits and side effects of therapy. Therapy goals should be distinguished between curative and palliative. Local treatments are mainly palliative and may add to quality of life. The advent of novel systemic therapies opens promising perspectives but its place in the therapeutic arsenal must be further determined.

## Introduction

Distant metastases in medullary thyroid carcinoma (MTC) are frequently present at initial presentation in sporadic MTC patients [1,2]. Organs most often affected include the lungs, bones and liver, and infrequently the brain. Distant metastases are frequently associated with persistent locoregional disease [3]. Patients with distant metastases at diagnosis have a poor prognosis, with only 40% surviving 10 years [2].

## Treatment goals

Treatment goals in advanced MTC are ultimately related to promoting the quality of life. It is important to explicitly discuss with the patient the expectations and goals with respect to quality of life in the individual context and to agree on a personalized treatment plan. In general, it is important to document the sites of metastases and the tumor volume as well as tumor progression to get an estimate of total tumor burden and prognosis. In addition, it is necessary to document signs and symptoms and estimate imminent problems. In patients with recurrent disease, an acceptable quality of life can usually be maintained for months or even years. Treatment decisions must therefore balance the usually slow progression rate and the reasonable life expectancy against the efficacy and side effects of therapies.

An important step is to decide whether any therapy will be curative or palliative, as the risk-benefit ratio of curative therapies is essentially different from palliative therapies. Curative therapies will *by definition* be aimed at improved survival and disease-free survival. The main objective of palliative therapies is to improve quality of life by relief of symptoms. When recurrent local disease is present or distant metastases are limited to a single organ curative surgical resection may be considered. Unfortunately, in advanced MTC, curative options are limited as both local recurrent disease and metastases are seldomly amenable to curative surgery. Active treatment is most often indicated in patients with lesions in critical locations such as brain metastases, threatening spinal cord compression, airway obstruction, bleeding, impending or active fracture or hormonal secretion.

## Documenting disease and disease progression

The discovery of metastases is often preceded by detectable or increasing serum levels of calcitonin or CEA. It must however be stressed that elevated levels of tumor markers are not a reason in itself to start therapies. Elevated tumor markers, and especially a low doubling time of serum calcitonin and CEA are rather considered risk factors for the presence and development of metastases and a poor prognosis [4,5]. Indeed, disease progression should always be confirmed by imaging before considering treatment. Patients who have elevated stable tumor markers without demonstrable tumor may have a good prognosis and quality of life [6-8]. Those patients are being followed with serum tumor marker measurements and regular imaging, the interval depending on the serum marker level and doubling time [1,4,5].

Imaging should identify all clinically relevant sites of disease, both for the identification of tumors that require local interventions and the selection of potential target lesions to assess response to systemic therapy. Imaging procedures in metastatic MTC include conventional contrast-enhanced spiral CT scan or MRI of the brain, neck, chest and liver and bone scintigraphy [3]. Diagnostic accuracy of Fluorodeoxyglucose (FDG)- PET scan is questionable [3,9]. F-DOPA-PET scintigraphy is promising and may provide additional information on tumor localization in patients with elevated tumor markers without evidence of disease on conventional imaging. However, the impact on clinical decision making needs to be confirmed [10].

In patients with measurable lesions, it should be evaluated if curative therapy is possible. When no curative options are present, disease progression must be documented. Usually, criteria for the initiation of systemic treatment are defined according to RECIST in combination with considerable tumor burden and/or symptoms.

## Local disease

Recurrent disease in the neck and mediastinum is frequently amenable to surgery, with either curative or palliative intent. Repeat surgery may normalize calcitonin levels in one third of patients and progression free survival may be fairly good, although no controlled studies have been performed. Surgical resection of locoregional recurrent MTC in patients without distant metastases should include compartmental dissection of disease in the neck compartments. In the presence of incurable advanced local or distant disease, less extensive neck surgery may be appropriate to avoid complications [11-13].

The role of external beam radiation therapy (EBRT) in recurrent local MTC is not clear. In patients with local disease in whom no curative surgery is possible, EBRT may improve locoregional disease control, although an improvement in overall survival has not been established [14].

## Distant metastases

Distant metastases limited to a single organ may be considered for curative surgical resection or another local treatment modality. Slow growing or stable metastases without clinical symptoms may be followed, the interval of imaging depending on the size of the tumor and the progression rate.

## Local treatment modalities for distant metastases

### Brain metastases

Clinically overt brain metastases from MTC are reported in 1–5% of MTC patients with local or metastatic disease [3]. Brain metastases are suspected in MTC patients with neurologic symptoms especially when distant metastases are present. Brain imaging should be performed in asymptomatic MTC patients before initiation of systemic therapy [15-17]. When small brain lesions are discovered, it may be decided to follow-up without treatment. In clinical studies in differentiated thyroid carcinoma and other tumors, it has been suggested that surgical resection in patients with solitary or a limited number of brain metastases may be associated with improved survival and quality of life [15-17]. Other treatment options include stereotactic radiosurgery or whole brain EBRT for clinically overt brain metastases.

### Bone metastases

Bone metastases occur in 45% of MTC patients with local or metastatic disease [3]. Small, asymptomatic bone metastases can be followed without treatment. Isolated bone metastases may be surgically resected. In some studies in differentiated thyroid carcinoma, it has been suggested that surgery is worthwhile when 5 or less bone metastases are present [18]. It is not known if these results can be expanded to MTC. EBRT in symptomatic bone lesions may lead to considerable reduction in pain in 80% of patients, which may last for months [14]. Experience from other tumors learns that symptomatic bone metastases can be successfully treated with percutaneous methods including cementoplasty, thermal ablation (radiofrequency or cryotherapy) and arterial embolization followed by surgery, or a combination of these methods, for pain reduction and bone consolidation [19,20]. Intravenous bisphosphonates are prescribed frequently for painful bony metastases in other malignancies, but this has not been investigated in MTC [21].

### Lung metastases

Lung metastases occur in 33% of MTC patients with local or metastatic disease [6]. In solitary lesions, surgical resection, either by open procedure or video assisted thoracoscopy can be considered. Alternatively, asymptomatic, stable or slowly progressive lesions can be followed. Lung or mediastinal lesions causing local compression of an airway or bleeding may be considered for surgery or EBRT, and lesions with central airway invasion may be amenable to the photodynamic therapy or airway stenting (when the tumor is >15 mm from the vocal cord) to improve quality of life [22]. Radiofrequency ablation may be indicated in patients with few ( <5,) predominantly peripheral lung metastases of < 40 mm in size.

### Liver metastases

Liver metastases occur in 45% of MTC patients with local or metastatic disease [6] . In large, progressive liver metastases or metastases associated with symptoms, local treatment is required. Treatment options include resection or percutaneous radiofrequency ablation. RFA may lead to prolonged symptom reduction in 90–95% of patients, including reduction of diarrhoea. Radiofrequency ablation is less effective in lesions > 50 mm [23,24].

## Systemic treatment

Conventional chemotherapy in patients with advanced MTC has limited efficacy, and considerable toxicity and is therefore not recommended [1].

In recent years, several kinase inhibitors have been evaluated in phase I and II clinical trials, including axitinib, cabozantinib (XL-184), lenvatinib (E7080), motesanib, pazopanib, sorafenib, sunitinib and vandetanib [25,26]. In phase II trials, several of these agents have demonstrated partial response rates in the range of 20–50% with a larger number of patients demonstrating prolonged stable disease. Two of these agents, vandetanib and cabozantinib have completed phase III clinical trials.

Vandetanib (300 mg/day) [27] prolonged median progression free survival from 19 months in the placebo arm to a predicted median of 31 months in the vandetanib arm; the improvement of pain and diarrhoea allowed a number of patients in the vandetanib arm to resume a normal social life. However, 12% of patients receiving vandetanib discontinued treatment due to toxicity and 35% required dose reduction because of an adverse event. Vandetanib was approved by the FDA in April 2011 and by the EMEA in February 2012. Cabozantinib (175 mg/day) prolonged PFS prolongation from 4 to 11 months and was approved by the FDA in November 2012 [28]. However, short-term toxicity of these therapies is significant, with dose reduction or treatment withdrawal in a significant proportion of patients; longterm toxicity needs to be investigated. There is currently no evidence for a higher treatment efficacy at an earlier than later stage when the tumor has progressed. This should lead to initiating these treatments only in patients with significant tumor burden and documented tumor progression.

## Other therapies

Therapies with immunization [29] and radiolabelled molecules have been performed in MTC sometimes with promising results. Treatment with 131-I-MIBG is generally regarded as ineffective for MTC [30].

## Treatment of hormonally active metastases

MTC is an endocrine active tumor and secretion of hormones other than calcitonin is frequently observed. Diarrhoea [31] is frequently present when tumor volume is high. Diarrhoea can be treated with anti-motility drugs including somatostatin analogues. When these therapies are not sufficient, local therapies to reduce tumor volume can be considered. Sometimes, CRH or ACTH production leads to Cushing syndrome, which may be a reason for local tumor reduction, cortisol synthesis inhibitors or bilateral adrenalectomy [32].

## List of abbreviations used

MTC: medullary thyroid carcinoma; CEA: carcinoembryonic antigen; EBRT: external beam radiation therapy.

## Competing interests

No conflict of interest.

## Declarations

This article has been published as part of *Thyroid Research* Volume 6 Supplement 1, 2013: European comments on Medullary Thyroid Cancer Management Guidelines of the American Thyroid Association. The full contents of the supplement are available online at http://www.thyroidresearchjournal.com/supplements/6/S1. Publication of this supplement has been supported by the European Thyroid Association-Cancer Research Network.
